# Timing and adequate attendance of antenatal care visits among women in Ethiopia

**DOI:** 10.1371/journal.pone.0184934

**Published:** 2017-09-18

**Authors:** Sanni Yaya, Ghose Bishwajit, Michael Ekholuenetale, Vaibhav Shah, Bernard Kadio, Ogochukwu Udenigwe

**Affiliations:** 1 School of International Development and Global Studies, Faculty of Social Sciences, University of Ottawa, Ottawa, Ontario, Canada; 2 School of Medicine and Health Management, Tongji Medical College, Huazhong University of Science and Technology, Wuhan, Hubei, China; 3 The Women’s Health and Action Research Centre, Benin City, Nigeria; 4 Interdisciplinary School Health Sciences, University of Ottawa, Ottawa, Ontario Canada; Boston University School of Public Health, UNITED STATES

## Abstract

**Introduction:**

Although ANC services are increasingly available to women in low and middle-income countries, their inadequate use persists. This suggests a misalignment between aims of the services and maternal beliefs and circumstances. Owing to the dearth of studies examining the timing and adequacy of content of care, this current study aims to investigate the timing and frequency of ANC visits in Ethiopia.

**Methods:**

Data was obtained from the nationally representative 2011 Ethiopian Demographic and Health Survey (EDHS) which used a two-stage cluster sampling design to provide estimates for the health and demographic variables of interest for the country. Our study focused on a sample of 10,896 women with history of at least one childbirth event. Percentages of timing and adequacy of ANC visits were conducted across the levels of selected factors. Variables which were associated at 5% significance level were examined in the multivariable logistic regression model for association between timing and frequency of ANC visits and the explanatory variables while controlling for covariates. Furthermore, we presented the approach to estimate marginal effects involving covariate-adjusted logistic regression with corresponding 95%CI of delayed initiation of ANC visits and inadequate ANC attendance. The method used involved predicted probabilities added up to a weighted average showing the covariate distribution in the population.

**Results:**

Results indicate that 66.3% of women did not use ANC at first trimester and 22.3% had ANC less than 4 visits. The results of this study were unique in that the association between delayed ANC visits and adequacy of ANC visits were examined using multivariable logistic model and the marginal effects using predicted probabilities. Results revealed that older age interval has higher odds of inadequate ANC visits. More so, type of place of residence was associated with delayed initiation of ANC visits, with rural women having the higher odds of delayed initiation of ANC visits (OR = 1.65; 95%CI: 1.26–2.18). However, rural women had 44% reduction in the odds of having inadequate ANC visits. In addition, multi-parity showed higher odds of delayed initiation of ANC visit when compared to the primigravida (OR = 2.20; 95%CI: 1.07–2.69). On the contrary, there was 36% reduction in the odds of multigravida having inadequate ANC visits when compared to the women who were primigravida. There were higher odds of inadequacy in ANC visits among women who engaged in sales/business, agriculture, skilled manual and other jobs when compared to women who currently do not work, after adjusting for covariates. From the predictive margins, assuming the distribution of all covariates remained the same among respondents, but everyone was aged 15–19 years, we would expect 71.8% delayed initiation of ANC visit. If everyone was aged 20-24years, 73.4%; 25-29years, 66.5%; 30-34years, 64.8%; 35-39years, 65.6%; 40-44years, 59.6% and 45-49years, we would expect 70.1% delayed initiation of ANC visit. If instead the distribution of age was as observed and for other covariates remained the same among respondents, but no respondent lived in the rural, we would expect about 61.4% delayed initiation of ANC visit; if however, everyone lived in the rural, and we would expect 71.6% delayed initiation in ANC visit. Model III revealed the predictive margins of all factors examined for delayed initiation for ANC visits, while Model IV presented the predictive marginal effects of the determinants of adequacy of ANC visits.

**Conclusion:**

The precise mechanism by which these factors affect ANC visits remain blurred at best. There may be factors on the demand side like the women’s empowerment, financial support of the husband, knowledge of ANC visits in the context of timing, frequency and the expectations of ANC visits might be mediating the effects through the factors found associated in this study. Supply side factors like the quality of ANC services, skilled staff, and geographic location of the health centers also mediate their effects through the highlighted factors. Irrespective of the knowledge about the precise mechanism of action, policy makers could focus on improving women’s empowerment, improving women’s education, reducing wealth inequity and facilitating improved utilization of ANC through modifications on the supply side factors such as geographic location and focus on hard to reach women.

## Introduction

Mortality from preventable pregnancy-related complications remains exceptionally high with three hundred thousand maternal deaths reported for 2015 [1]. According to the Maternal Mortality Estimation Inter-agency Group, developing countries accounted for approximately 99% of global maternal deaths in 2015 with 66% of these deaths occurring in sub-Saharan Africa [2]. Similarly, a recent Ethiopian Survey indicates a MMR value of 412 [3]. Moreover, despite Ethiopia’s 71.8% decline in MMR between 1990 and 2015, the lifetime risk of maternal death remains alarmingly high with 1 in 64 women at risk of dying from maternal causes, taking into account other competing causes of death [2,4]. Women across every cultural and socio-demographic backgrounds require a positive pregnancy experience and high quality antenatal and postnatal care [5].

Antenatal care (ANC) from skilled providers at health facilities is a priority in ensuring and enriching maternofetal health. More importantly, timely and appropriate ANC practices has life-saving potential for mother and child [6,7]. The benefits of ANC visits include nutrition and health checks, opportunity to detect pregnancy risks, counselling and support for women and their families, and a higher likelihood of delivery in the presence of skilled birth attendants leading to lower maternofetal deaths [8–11]. Citing increased foetal death and reduced maternal satisfaction of the previous ANC focused model, the World Health Organisation (WHO) recently released updated clinical guidelines for ANC services[12]. Notable updates include the five critical maternofetal interventions during ANC visits: nutritional interventions, maternal and foetal assessments, preventive measures such as vaccinations, interventions for common physiological symptoms, and interventions for improving utilization of care [6]. The previously recommended ANC model of four visits was increased to eight visits for the duration of the pregnancy with the first contact occurring within the first 12 weeks (gestational period) and subsequent ones occurring at 4 week intervals [6,12].

Timely ANC is generally acknowledged to be an effective method of preventing adverse pregnancy outcomes. Timing of first ANC visit has been shown to predict the provision of interventions recommended by WHO [13–15]. A recent study in Pakistan reported that women whose first ANC visit occurred within the first 12 weeks of pregnancy received the full range of WHO-recommended content of care. However, women whose first ANC visits occurred late during pregnancy were less likely to receive the full range of services. A recent study in Northwest Ethiopia reported that 52% of participants (n = 369) booked their first ANC visit after 4 months of pregnancy [16]. A similar study in Central Ethiopia reported less prevalence with 26% of 446 pregnant women. Women under 30 years old who have formal education, with previous positive experience of early ANC services, were more likely to utilize ANC services early in their pregnancy. On the contrary, other studies demonstrated that a history of early ANC visit is not an indicator of early utilization of ANC services in current pregnancy, and that previous experiences with ANC service providers and pregnancy intention determined early service utilization [17,18].

A long-standing assumption has been that the availability of a high quality and minimal cost program will encourage its adequate use [19]. Although ANC services are increasingly available to women in low and middle income countries, their inadequate use persists [18,19]. This suggests a misalignment between aims of the services and maternal beliefs and circumstances. Studies examining the prevalence of ANC utilizations in Ethiopia revealed inadequate ANC visits with few pregnant women receiving ANC services four or more times [3,20,21]. Maternal wealth, knowledge of the recommended number of visits, and beliefs toward pregnancy positively impacted maternal adherence to the recommended ANC visits [22].The use of ANC, irrespective of the number of visits, was highest among younger, low parity and literate mothers. The highest use of ANC services also occurred among mothers with a high exposure to the media and mothers whose pregnancy was planned [20,23]. Furthermore, the importance of husband/partner’s involvement in maternal health has been emphasised to increase maternal use of ANC services [19,20,24,25].

In order for ANC to be deemed effective, the timing and adequacy of content of care should be examined [15]. There is a dearth of this assessment in studies conducted with pregnant women in Ethiopia. Therefore, the objective of this study was to assess timing and adequate attendance of ANC visit among women with experience of motherhood in Ethiopia.

## Methods

### Data source

This study used secondary data from the 2011 Ethiopian Demographic and Health Survey (EDHS). We accessed the data from MEASURE DHS database at http://dhsprogram.com/data/available-datasets.cfm. The Ethiopia survey was conducted by the Ethiopian Central Statistical Agency as part of the International Demographic and Health Survey program known as MEASURE DHS, which is currently active in 90 countries and conducted under the auspices of the United States Agency for International Development (USAID) with the technical assistance of ICF International, based in the USA. The Demographic and Health Surveys (DHSs) are free, public datasets, though researchers must register with MEASURE DHS and submit a request before access to DHS data is granted. This data request system ensures that all users understand and agree to basic data usage ethics standards. Sampling procedures were published in the final report [26].

### Sampling procedures

The 2011 EDHS samples were selected using a stratified, two-stage cluster sampling design to provide estimates for the health and demographic variables of interest for the country. The sampling frame consists of a total of 85,057 Enumeration Areas (EAs). A nationally representative sample of 17,817 households was included in data collection.

### Variables

The outcome variables of this study were- 1) Timing of first ANC attendance, and 2) Total number of ANC attendance. ANC visits are of critical important to avert pregnancy related complications, counselling for maternal and foetal health, preparedness for health-facility delivery [27]. WHO recommends the first ANC visit should take place within the first trimester of gestation, and at least four visits during the course of the pregnancy. According to these guidelines, the outcome variables are categorized as: 1) Timing of first ANC attendance (Within 3 months of gestation = early, and beyond 3 month = delayed), and 2) Total number of ANC attendance (<4 visits and 4 or more visits).

Besides these, several individual and community level factors were considered as explanatory variables for their relevance in the uptake of ANC care. These were: Age (15–19, 20–24, 25–29, 30–34, 35–39, 40–44, 45–49), Type of place of residence (Urban, Rural), Highest educational level (Nil, Primary, Secondary, Higher), Wealth index (Poorest, Poorer, Middle, Richer, Richest), Occupation (Not working, Sales, Agricultural, Other Skilled manual), Frequency of reading newspaper or magazine (Not at all, Less than once a week, At least once a week), Frequency of listening to radio (Not at all, Less than once a week, At least once a week), Frequency of watching television (Not at all, Less than once a week, At least once a week), Sex of household head (Male, Female), Decision maker of respondent's health care (Respondent alone, Respondent and husband/partner together, Husband/partner alone).

### Analytical procedure

Data analyses were carried out using STATA 14. The dataset was checked for cases which fulfilled all the inclusion criteria: age being 15 years and above, having experienced at least one childbirth, availability of information on ANC visits. Basic characteristics of the participants were tabled using frequencies and percentages. Chi-square test was performed to examine the association between timing and frequency of ANC visits and the explanatory variables. Furthermore, multivariable logistic regression analysis was used to determine the odds ratios (with corresponding 95%CI) of delayed initiation of ANC visits and less than four ANC visits. Examining marginal effects, we explored the disparities in predicted probabilities across the factors, in which estimated effects were proportionately adjusted according to a weight for each level of the covariates. Based on the estimation of marginal effects, we predicted the probability of delayed initiation and inadequacy of ANC visits [28]. Thus;

$$\mathrm{Pr}(Y=1|\mathrm{Set}[E=e])=\sum_{z}{\hat{p}}_{ez}\mathrm{Pr}(Z=z);$$

Where Set[E = e] reflects putting all observations to a single exposure level e, and Z = z refers to a given set of observed values for the covariate vector Z. Furthermore, ${\hat{p}}_{ez}$ is the predicted probabilities of delayed initiation of ANC visits and adequacy of ANC visits respectively for any E = e and Z = z. The marginal effects indicate a weighted average over the distribution of the covariates or confounders and are equal to estimates got by standardizing to the entire population. As a post logistic regression test, the exposure *E* is set to the level *e* for all respondents in the dataset, and the logistic regression coefficients are used to compute predicted probabilities for every respondent at their observed covariate pattern and newly exposure value. Since predicted probabilities are computed under the same distribution of *Z*, there is no covariate of the corresponding effect measure estimates. After obtaining results of the logistic regression model; the margins command was then used to compute the marginal effects of the factors in STATA [28].

### Ethics statement

Before each interview, all participants gave informed consent to take part in the survey. The DHS Program maintains strict standards for ensuring data anonymity and protecting the privacy of all participants. ICF International ensures that the survey complies with the U.S. Department of Health and Human Services regulations for the protection of human subjects, whilst the host country ensures that the survey complies with local laws and norms. Further approval for this study was not required since the data is secondary and is available in the public domain. More details regarding DHS data and ethical standards are available at: http://goo.gl/ny8T6X.

## Results

### Background characteristics of individual women

In total 10,896 women with history of at least one childbirth event were included in the study. Table 1 shows that about a quarter (24.5%) of them were in the age group of 25–29 years and about three-quarter (74.6%) were of rural origin. About two-third (65.8%) had no formal education, and 26.1% had primary level education. Most of the women were from the households in the richest wealth quintile (27.1%), and about half had no employment (47.9%). Regarding paper and electronic media use status, radio was the most popular one with 18.4% women reported using it at least once a week, followed by TV (16.3% watched at least once a week) and newspaper (2.7% read at least once a week). More than three-quarter (75.2%) of the households were male-headed, and in 56.5% the cases respondent’s healthcare decisions were made together with husband/partner. A prominent number of the women were married (57.4%), while estimated 81.5% were multiparous. Approximately one-tenth of the respondents have terminated at least one pregnancy, and about 25.8% (later = 17.2 and no more = 8.6) had unwanted pregnancies.

**Table 1:** Women reporting first ANC visit during first trimester and attending at least 4 ANC visits.

Variable	n(%)	First ANC Visit		P	Frequency of ANC visits		p
		Within 1^st^ trimester (33.7)	Delayed (66.3)		≥4 (77.7)	<4 (22.3)	
**Age in 5-year groups**							
15–19	421(3.9)	27.7	72.3	.012*	83.4	16.6	< .0001*
20–24	1674(15.4)	30.9	69.1		75.9	24.1	
25–29	2665(24.5)	36.1	63.9		75.9	24.1	
30–34	1937(17.8)	35.4	64.6		78.1	21.9	
35–39	1860(17.1)	32.4	67.6		78.1	21.9	
40–44	1274(11.7)	32.0	68.0		83.8	16.2	
45–49	1065(9.8)	18.0	82.0		88.9	11.1	
**Type of place of residence**				< .0001*			< .0001*
Urban	2767(25.4)	51.9	48.1		44.0	56.0	
Rural	8129(74.6)	23.5	76.5		86.1	13.9	
**Highest educational level**				< .0001*			< .0001*
No education	7166(65.8)	25.4	74.6		87.0	13.0	
Primary	2849(26.1)	35.4	64.6		67.5	32.5	
Secondary	556(5.1)	54.0	46.0		29.2	70.8	
Higher	325(3.0)	65.5	34.5		20.8	79.2	
**Wealth index**				< .0001*			< .0001*
Poorest	2882(26.5)	25.7	74.3		92.4	7.6	
Poorer	1761(16.2)	19.1	80.9		88.5	11.5	
Middle	1637(15.0)	20.2	79.8		84.7	15.3	
Richer	1663(15.3)	25.3	74.7		77.7	22.3	
Richest	2953(27.1)	50.4	49.6		44.5	55.5	
**Occupation**				< .0001*			< .0001*
Not working	5217(47.9)	34.4	65.6		80.9	19.1	
Sales	1844(16.9)	35.3	64.7		69.5	30.5	
Agricultural	2284(21.0)	19.0	81.0		84.5	15.5	
Other	656(6.0)	55.0	45.0		44.5	55.5	
Skilled manual	895(8.2)	35.0	65.0		76.0	24.0	
**Frequency of reading newspaper or magazine**				< .0001*			< .0001*
Not at all	9621(88.3)	30.2	69.8		81.8	18.2	
Less than once a week	976(9.0)	42.3	57.7		43.6	56.4	
At least once a week	299(2.7)	64.5	35.5		29.2	70.8	
**Frequency of listening to radio**				< .0001*			< .0001*
Not at all	5764(52.9)	28.9	71.1		85.7	14.3	
Less than once a week	3127(28.7)	31.9	68.1		73.5	26.5	
At least once a week	2005(18.4)	42.9	57.1		59.8	40.2	
**Frequency of watching television**				< .0001*			< .0001*
Not at all	6835(62.7)	24.7	75.3		87.9	12.1	
Less than once a week	2283(21.0)	30.1	69.9		69.9	30.1	
At least once a week	1778(16.3)	55.2	44.8		37.7	62.3	
**Sex of household head**				< .0001*			.006*
Male	8189(75.2)	31.0	69.0		78.7	21.3	
Female	2707(24.8)	41.6	58.4		75.0	25.0	
**Decision maker of respondent's health care** *				.007*			< .0001*
Respondent alone	1574(17.1)	38.2	61.8		69.7	30.3	
Respondent and husband/partner	5201(56.5)	31.0	69.0		76.4	23.6	
Husband/partner alone	2434(26.4)	30.8	69.2		86.4	13.6	
**Wanted pregnancy**				.312			.772
Then	5760(74.2)	33.8	66.2		78.1	21.9	
Later	1335(17.2)	30.6	69.4		76.8	23.2	
No more	664(8.6)	37.9	62.1		77.7	22.3	
**Region**				.223			< .0001*
Tigray	1728(10.5)	32.4	67.6		79.3	20.7	
Affar	1291(7.8)	32.0	68.0		81.9	18.1	
Amhara	2087(12.6)	38.1	61.9		73.1	26.9	
Oromiya	2135(12.9)	34.5	65.5		75.6	24.4	
Somali	914(5.5)	30.3	69.7		73.6	26.4	
Benishangul-Gumuz	1259(7.6)	31.0	69.0		76.9	23.1	
SNNP	2034(12.3)	31.0	69.0		78.0	22.0	
Gambela	1130(6.8)	30.4	69.6		82.2	17.8	
Harari	1101(6.7)	35.0	65.0		79.2	20.8	
Addis Ababa	1741(10.5)	30.2	69.8		83.0	17.0	
Dire Dawa	1095(6.6)	38.1	61.9		78.5	21.5	
**Ever had a terminated pregnancy**				.193			.211
Yes	1428(8.7)	30.0	70.0		76.1	23.9	
No	15079(91.3)	33.6	66.4		78.1	21.9	
**Current marital status**				.070			.076
Never in union	4413(26.7)	31.1	68.9		79.7	20.3	
Married	9478(57.4)	33.4	66.6		76.9	23.1	
Living with partner	726(4.4)	28.0	72.0		81.3	18.7	
Widowed	581(3.5)	41.7	58.3		78.0	22.0	
Divorced	922(5.6)	38.5	61.5		77.3	22.7	
No longer living together/separated	395(2.4)	36.8	63.2		81.0	19.0	
**Total children ever born**				<0.001*			<0.001*
Primi	2015(18.5)	41.8	58.2		65.5	34.5	
Multi	8881(81.5)	30.4	69.6		80.9	19.1	

*significant at p<0.05.

The test of association revealed that age, type of place of residence, educational level, wealth index, occupation, use of media (reading newspaper or magazine, listening to radio and watching television), sex of household head, decision maker of respondents’ health, region and parity were associated with first ANC visits and frequency of ANC visits respectively. In sum, the variables which were associated with both dependent variables were selected into the regression model to generate the marginal effects in Table 2.

**Table 2:** Logistic regression of the factors associated with delayed initiation of ANC visits and frequency of ANC visits among women of reproductive age in Ethiopia.

Variable	Delayed initiation of ANC visit (Model I)			ANC visits <4 (Model II)		
	Odds ratio	95% CI	P-value	Odds ratio	95% CI	P-value
**Age in 5-year groups**						
15–19	1.0			1.0		
20–24	1.10	0.73–1.66	0.648	1.47	1.04–2.08	0.030*
25–29	0.75	0.49–1.15	0.185	1.82	1.27–2.61	0.001*
30–34	0.69	0.44–1.08	0.107	2.10	1.43–3.07	<0.001*
35–39	0.72	0.45–1.14	0.164	2.43	1.65–3.58	<0.001*
40–44	0.53	0.31–0.90	0.020*	2.07	1.33–3.21	0.001*
45–49	0.91	0.41–2.03	0.821	1.74	0.96–3.14	0.068
**Type of place of residence**						
Urban	1.0			1.0		
Rural	1.65	1.26–2.18	<0.001*	0.56	0.45–0.71	<0.001*
**Highest educational level**						
No education	1.0			1.0		
Primary	0.79	0.64–0.96	0.017*	1.77	1.51–2.08	<0.001*
Secondary	0.62	0.43–0.88	0.007*	3.41	2.39–4.87	<0.001*
Higher	0.45	0.28–0.74	0.002*	2.96	1.77–4.94	<0.001*
**Wealth index**						
Poorest	1.0			1.0		
Poorer	1.32	0.95–1.82	0.095	1.44	1.12–1.84	0.004*
Middle	1.23	0.90–1.67	0.201	1.91	1.51–2.42	<0.001*
Richer	1.00	0.75–1.34	0.990	2.45	1.95–3.09	<0.001*
Richest	0.73	0.52–1.03	0.072	3.79	2.87–5.02	<0.001*
**Occupation**						
Not working	1.0			1.00		
Sales	1.09	0.88–1.36	0.441	1.26	1.05–1.52	0.014*
Agricultural	1.37	1.06–1.77	0.017*	1.26	1.04–1.51	0.016*
Other	0.86	0.61–1.23	0.420	1.36	0.97–1.92	0.077
Skilled manual	0.97	0.70–1.35	0.872	1.38	1.05–1.80	0.019*
**Frequency of reading newspaper or magazine**						
Not at all	1.0			1.0		
Less than once a week	1.37	1.04–1.82	0.027*	1.13	0.87–1.46	0.351
At least once a week	0.78	0.50–1.21	0.270	1.31	0.81–2.11	0.269
**Frequency of listening to radio**						
Not at all	1.0			1.0		
Less than once a week	1.00	0.82–1.22	0.983	1.25	1.06–1.47	0.007*
At least once a week	0.93	0.74–1.17	0.540	1.29	1.06–1.56	0.010*
**Frequency of watching television**						
Not at all	1.0			1.0		
Less than once a week	1.11	0.89–1.39	0.358	1.59	1.34–1.89	<0.001*
At least once a week	0.64	0.49–0.83	0.001*	2.29	1.82–2.88	<0.001*
**Sex of household head**						
Male	1.0			1.0		
Female	0.86	0.69–1.07	0.181	0.94	0.77–1.14	0.508
**Decision maker of respondent's health care** *						
Respondent alone	1.0			1.0		
Respondent and husband/partner	0.99	0.80–1.23	0.927	1.05	0.87–1.26	0.646
Husband/partner alone	0.78	0.60–1.01	0.064	0.79	0.63–0.99	0.037*
**Region**						
Tigray	-	-	-	1.0		
Affar	-	-	-	1.13	0.81–1.58	0.477
Amhara	-	-	-	1.29	0.96–1.72	0.086
Oromiya	-	-	-	0.98	0.74–1.30	0.898
Somali	-	-	-	1.22	0.85–1.77	0.287
Benishangul-Gumuz	-	-	-	0.95	0.70–1.31	0.773
SNNP	-	-	-	1.09	0.82–1.44	0.563
Gambela	-	-	-	1.09	0.74–1.60	0.654
Harari	-	-	-	0.955	0.66–1.38	0.807
Addis Ababa	-	-	-	1.07	0.78–1.47	0.690
Dire Dawa	-	-	-	1.05	0.73–1.50	0.805
**Parity**						
Primi	1.0			1.0		
Multi	2.20	1.07–2.69	0.012*	0.64	0.52–0.79	<0.001*

*significant at p<0.05; N.B.

Reference category were: Timing of ANC within 1st trimester, and attending at least 4 ANC visits. Pseudo R-squared: Model I = 0.235; Model II = 0.105

### Logistic regression analysis

From the logistic regression model, age was significantly associated frequency of ANC visits. Results revealed that older age interval has higher odds of inadequate ANC visits. More so, type of place of residence was associated with delayed initiation of ANC visits, with rural women having the higher odds of delayed initiation of ANC visits (OR = 1.65; 95%CI: 1.26–2.18). However, rural women had 44% reduction in the odds of having inadequate ANC visits. Furthermore, higher educational level had reduction in the odds of delayed initiation of ANC visits when compared to women with no formal education after controlling for confounders (see Table 2), while higher educational attainment had higher odds of inadequate ANC visits when compared to women without formal education. For wealth index, it was found that respondents of high economic class had higher odds of inadequate ANC visits. In addition, multi-parity showed higher odds of delayed initiation of ANC visit when compared to the primigravida (OR = 2.20; 95%CI: 1.07–2.69). On the contrary, there was 36% reduction in the odds of multigravida having inadequate ANC visits when compared to the women who were primigravida. There were higher odds of inadequacy in ANC visits among women who engage in sales/business, agriculture, skilled manual and other jobs when compared to women who currently do not work, after adjusting for covariates. Also, women who are engage in agricultural occupation were 1.37 times more likely to have delayed initiation in ANC when compared to women who currently do not work, after controlling for covariates (OR = 1.37; 95%CI: 1.06–1.77), See Table 2.

### Marginal effect analysis

In Table 3 margins was conducted to decipher the effects of the factors associated with delayed initiation of ANC and adequacy of ANC visits. From the predictive margins, assuming the distribution of all covariates remained the same among respondents, but everyone was aged 15-19years, we would expect 71.8% delayed initiation of ANC visit. If everyone were aged 20-24years, 73.4%; 25-29years, 66.5%; 30-34years, 64.8%; 35-39years, 65.6%; 40-44years, 59.6% and 45-49years, we would expect 70.1% delayed initiation of ANC visit. If instead the distribution of age was as observed and for other covariates remained the same among respondents, but no respondent lived in the rural, we would expect about 61.4% delayed initiation of ANC visit; if however, everyone lived in the rural, and we would expect 71.6% delayed initiation in ANC visit. In addition, if instead the distribution of age and place of residence were as observed and other covariates remained the same in the respondents, but everyone had no formal education, we would expect 71.2% delayed initiation in ANC visit; similarly, we would expect 66.5%, 61.5% and 54.7% delayed initiation in ANC for primary, secondary and tertiary educational level respectively.

**Table 3:** Marginal effect of the factors associated with delayed initiation of ANC visits and frequency of ANC visits among women of reproductive age in Ethiopia.

Variable	Delayed initiation of ANC visit (Model III)			ANC visits <4 (Model IV)		
	Marginal effect	95% CI	P-value	Marginal effect	95% CI	P-value
**Age in 5-year groups**						
15–19	0.718	0.649–0.786	<0.001*	0.153	0.122–0.185	<0.001*
20–24	0.734	0.703–0.766	<0.001*	0.193	0.175–0.211	<0.001*
25–29	0.665	0.637–0.693	<0.001*	0.219	0.204–0.234	<0.001*
30–34	0.648	0.611–0.686	<0.001*	0.236	0.216–0.257	<0.001*
35–39	0.656	0.614–0.699	<0.001*	0.256	0.232–0.279	<0.001*
40–44	0.596	0.527–0.666	<0.001*	0.234	0.200–0.269	<0.001*
45–49	0.701	0.576–0.826	<0.001*	0.213	0.155–0.279	<0.001*
**Type of place of residence**						
Urban	0.614	0.573–0.655	<0.001*	0.278	0.249–0.308	<0.001*
Rural	0.716	0.690–0.742	<0.001*	0.197	0.185–0.209	<0.001*
**Highest educational level**						
No education	0.712	0.686–0.737	<0.001*	0.180	0.168–0.192	<0.001*
Primary	0.665	0.638–0.692	<0.001*	0.258	0.240–0.276	<0.001*
Secondary	0.615	0.551–0.679	<0.001*	0.370	0.307–0.432	<0.001*
Higher	0.547	0.447–0.646	<0.001*	0.344	0.254–0.433	<0.001*
**Wealth index**						
Poorest	0.692	0.647–0.738	<0.001*	0.131	0.112–0.149	<0.001*
Poorer	0.743	0.695–0.792	<0.001*	0.170	0.146–0.193	<0.001*
Middle	0.730	0.684–0.777	<0.001*	0.206	0.182–0.230	<0.001*
Richer	0.693	0.650–0.735	<0.001*	0.242	0.219–0.266	<0.001*
Richest	0.629	0.590–0.668	<0.001*	0.316	0.281–0.350	<0.001*
**Occupation**						
Not working	0.667	0.644–0.690	<0.001*	0.203	0.192–0.214	<0.001*
Sales	0.684	0.648–0.719	<0.001*	0.232	0.211–0.253	<0.001*
Agricultural	0.726	0.685–0.766	<0.001*	0.231	0.211–0.251	<0.001*
Other	0.638	0.571–0.705	<0.001*	0.242	0.198–0.286	<0.001*
Skilled manual	0.662	0.602–0.722	<0.001*	0.243	0.210–0.277	<0.001*
**Frequency of reading newspaper or magazine**						
Not at all	0.670	0.652–0.688	<0.001*	0.216	0.207–0.225	<0.001*
Less than once a week	0.728	0.686–0.769	<0.001*	0.231	0.200–0.263	<0.001*
At least once a week	0.621	0.535–0.707	<0.001*	0.251	0.186–0.316	<0.001*
**Frequency of listening to radio**						
Not at all	0.680	0.655–0.706	<0.001*	0.202	0.190–0.215	<0.001*
Less than once a week	0.681	0.654–0.708	<0.001*	0.231	0.215–0.246	<0.001*
At least once a week	0.667	0.634–0.700	<0.001*	0.234	0.214–0.255	<0.001*
**Frequency of watching television**						
Not at all	0.697	0.670–0.723	<0.001*	0.183	0.171–0.196	<0.001*
Less than once a week	0.717	0.685–0.748	<0.001*	0.245	0.225–0.265	<0.001*
At least once a week	0.605	0.563–0.647	<0.001*	0.303	0.270–0.336	<0.001*
**Sex of household head**						
Male	0.682	0.665–0.698	<0.001*	0.219	0.210–0.228	<0.001*
Female	0.653	0.613–0.692	<0.001*	0.211	0.190–0.233	<0.001*
**Decision maker of respondent's health care** *						
Respondent alone	0.688	0.653–0.722	<0.001*	0.222	0.201–0.243	<0.001*
Respondent and husband/partner	0.686	0.666–0.706	<0.001*	0.228	0.216–0.239	<0.001*
Husband/partner alone	0.640	0.605–0.675	<0.001*	0.193	0.177–0.210	<0.001*
**Region**						
Tigray	-	-	-	0.210	0.183–0.237	<0.001*
Affar	-	-	-	0.225	0.193–0.256	<0.001*
Amhara	-	-	-	0.242	0.218–0.266	<0.001*
Oromiya	-	-	-	0.207	0.186–0.229	<0.001*
Somali	-	-	-	0.235	0.197–0.273	<0.001*
Benishangul-Gumuz	-	-	-	0.204	0.178–0.230	<0.001*
SNNP	-	-	-	0.220	0.198–0.242	<0.001*
Gambela	-	-	-	0.220	0.182–0.259	<0.001*
Harari	-	-	-	0.204	0.170–0.238	<0.001*
Addis Ababa	-	-	-	0.218	0.190–0.246	<0.001*
Dire Dawa	-	-	-	0.215	0.181–0.249	<0.001*
**Parity**						
Primi	0.633	0.594–0.672	<0.001*	0.266	0.240–0.292	<0.001*
Multi	0.691	0.672–0.710	<0.001*	0.207	0.198–0.217	<0.001*

STATA command used to obtain the results of marginal effect: margins age residence education wealth occupation newspaper radio TV sex_household_head decision_maker region parity

*significant at p<0.05.

Model III in Table 3 revealed the predictive margins of all factors examined for delayed initiation for ANC visits. Furthermore, Model IV presented the predictive marginal effects of the determinants of adequacy of ANC visits. The results showed that if the spread of other covariates remained equal in the population, while everyone was aged 15-19years, we would expect 15.3% inadequate ANC visits, if everyone was aged 20-24years, we would expect 19.3% inadequate ANC visits, and so on for other age intervals (see Model IV). If as an alternative, the dispersal of all age intervals were as observed but all respondents lived in the urban area, then we expect 27.8% inadequate ANC visits and for rural areas, we would expect 19.7% ANC visits <4. Several factors were examined in Model III and IV, Table 3; which include age, type of place of residence, educational attainment, wealth index, occupation, frequency of reading newspaper or magazine, listening to radio, watching television, sex of household head, decision maker of respondent’s health care, region and parity. In Model III and IV, we practically obtained the predictive margins of the factors of delayed initiation of ANC visits and inadequate ANC visits.

## Discussion

As stated by WHO, a pregnant woman should commence ANC in the first trimester. Nonetheless, a large number of women from developing countries do not utilize ANC according to the guidelines. To meet international standards, the timing and number of ANC visits are vital in the detection and management of pregnancy complications [29]. Timing of the first ANC visit is correlated to ANC adequacy. WHO recommended that women should start ANC early, at first trimester to have at least four ANC visits [6]. Early commencement of ANC makes women to have sufficient number of visits and adequate services [30] to know probable complications during pregnancy and introduce suitable treatment. The results of this study revealed that an estimated 66.3% of women had delayed first ANC visits, while about one-quarter of the women utilize ANC less than four (<4) times. This clearly showed that there is still under-utilization of ANC and consequently the possible reason for high maternal mortality. In the test of association, age, type of residence i.e. urban vs rural, educational level of mothers, wealth index, occupation, use of media (reading of newspaper, listening to radio and watching of television), sex of household head, decision maker of respondent’s healthcare, region and total children ever born were associated with initiation of ANC visits and frequency of ANC visits.

Age was associated to delayed initiation of ANC visits and less than 4 ANC visits. In other words, extreme age categories of the mother behaved similar. Mixed results were found in previous studies in the context of association of age of the mother with ANC visits, both in terms of timing and frequency. A previous study indicated that late attendance at ANC visits and utilization of less than 4 ANC visits were associated with younger age group of mothers [31]. More so, findings similar to the current study and indicated that age of the mother at delivery was significantly associated with timing and frequency of ANC visits [32]. However, beyond the scope of the present study, it would be interesting to find the underlying factors mediating the effect of age of mother on initiation of ANC visits and completing the required number of ANC visits (at least 4). Previous studies have identified factors such as lack of knowledge of the timing and importance of ANC visits. Presence of unwanted pregnancies have also been cited as a factor behind delayed initiation of ANC visits and sometimes even to the extent of not utilizing ANC visits at all [32].

In terms of type of residence i.e. urban vs rural, those women residing in rural areas had more effect in delayed ANC visits. This finding is consistent with previous studies [31,32,33]. The supply factors such as quality of ANC services provided and distance of the healthcare facility might be mediating such results. Other factors like knowledge about the importance of timing and frequency of ANC visits and costs associated with ANC visits have also been cited. One previous study has specifically focussed on the need to sensitize rural areas about the importance of timing and frequency of ANC visits. The study also highlighted the importance of factors such as husband’s support in ANC visits particularly in provision of financial support [34].

Education was also found correlated with timing of ANC visits and completing the required number of ANC visits. Higher educational level implied that the women will register early for ANC visits and complete at least 4 ANC visits. This might be directly related to the knowledge about the importance of ANC visits gained through formal education. However, factors such as increased autonomy for women, more access to health education and improved financial status due to possibly increased employment might all be of importance. For example, one previous study has specifically concluded to focus on educating girls beyond secondary level and increase media penetration of the importance of ANC visits [35].

Wealth inequity was another factor affecting ANC visits in terms of timing and frequency. Although the difference was not uniform, the poorest were more likely than other categories to have delayed initiation of ANC visits and less than the minimum required ANC visits.

Decision making authority of the women was another variable found to be associated with timing and frequency of ANC visits. Surprisingly when women alone were decision makers the likelihood of delayed initiation of ANC visits was more compared to when the decision was made jointly with the husband or by husband alone. This may be a depiction of low women’s empowerment. It could also be a result of financial dependence on husband. However, paradoxically when women alone were the decision maker, she was more likely to complete at least 4 ANC visits when compared with joint decision making with the husband or decision by the husband alone. Previous studies have highlighted the importance of improving women’s empowerment particularly in the aspect of health decision-making and also focus on paternal education to reduce the magnitude of the problem [34]. The findings one another study are mixed particularly when women’s empowerment and male accompaniment to antenatal visits was considered [36]. However, studies have also shown that the expectations of ANC visits and the costs associated with it might be deterrent when husband was the decision maker [34].

Furthermore, the inverse correlation in the categorical independent variables between timing and adequacy of ANC visits was also noticeable in occupation, use of media such as reading magazines/newspaper, listening to radio and watching television and parity. Overall age of the mother, mother’s level of education, type of residence i.e. urban vs rural, wealth inequity, decision making authority of the mother, occupation, use of media (newspaper, radio and television), sex of household head, region and parity were all associated with timing and frequency of ANC visits. Intricately related associations are a possibility and there may be factors both at the demand side as well as supply-side which mediate the effects of these identified factors.

## Conclusions

This study has become the foremost to provide the marginal effects of the factors associated with timing and adequacy of ANC visits among women of reproductive age in Ethiopia, and has provided great improvement of reporting absolute risk over the conventional measures of association. The study concludes that age of the mother, education level of the mother, type of residence i.e. urban vs rural, decision making authority of the mother amongst others are associated with the timing and frequency of ANC visits. The precise mechanism by which these factors affect ANC visits remain blurred at best. There may be factors on the demand side like the women’s empowerment, financial support of the husband, knowledge of ANC visits in the context of timing, frequency and the expectations of ANC visits might be mediating the effects through the factors found associated in this study. Supply side factors like the quality of ANC services, skilled staff, geographic location of the health centers also mediate their effects through the highlighted factors. Further research may be required to decipher the exact mechanism through which factors such as age of the mother, educational level and type of residence i.e. urban vs rural affect ANC visits both in terms of timing and frequency. However irrespective of the knowledge about the precise mechanism of action, policy makers could focus on improving women’s empowerment, improving women’s education, reducing wealth inequity and facilitating improved utilization of ANC through modifications on the supply side factors such as geographic location and focus on hard to reach women. These structural changes in policy can indubitably have wider ramifications than merely improving ANC visits.
